# The Gammaherpesvirus m2 Protein Manipulates the Fyn/Vav Pathway through a Multidocking Mechanism of Assembly

**DOI:** 10.1371/journal.pone.0001654

**Published:** 2008-02-27

**Authors:** Marta Pires de Miranda, Marta Alenquer, Sofia Marques, Lénia Rodrigues, Filipa Lopes, Xosé R. Bustelo, J. Pedro Simas

**Affiliations:** 1 Instituto de Microbiologia e Instituto de Medicina Molecular, Faculdade de Medicina, Universidade de Lisboa, Lisboa, Portugal; 2 Instituto Gulbenkian de Ciência, Oeiras, Portugal; 3 Centro de Investigación del Cáncer and Instituto de Biología Molecular y Celular del Cáncer, Consejo Superior de Investigaciones Científicas (CSIC), University of Salamanca, Salamanca, Spain; Institut Pasteur, France

## Abstract

To establish latent infections in B-cells, gammaherpesviruses express proteins in the infected B-cells of the host that spuriously activate signalling pathways located downstream of the B-cell receptor. One such protein is M2, a murine gammaherpesvirus 68-encoded molecule that activates the Vav1/Rac1 pathway via the formation of trimolecular complexes with Scr family members. Previous reports have shown that the formation of this heteromolecular complex involves interactions between a proline rich region of M2 and the Vav1 and Fyn SH3 domains. Here, we show that the optimal association of these proteins requires a second structural motif encompassing two tyrosine residues (Tyr120 and 129). These residues are inducibly phosphorylated by Fyn in non-hematopoietic cells and constitutively phosphorylated in B-cells. We also demonstrate that the phosphorylation of Tyr120 creates specific docking sites for the SH2 domains of both Vav1 and Fyn, a condition *sine qua non* for the optimal association of these two signalling proteins in vivo. Interestingly, signaling experiments indicate that the expression of M2 in B-cells promotes the tyrosine phosphorylation of Vav1 and additional signaling proteins, a biological process that requires the integrity of both the M2 phosphotyrosine and proline rich region motifs. By infecting mice with viruses mutated in the *m2* locus, we show that the integrity of each of these two M2 docking motifs is essential for the early steps of murine gammaherpesvirus-68 latency. Taken together, these results indicate that the M2 phosphotyrosine motif and the previously described M2 proline rich region work in a concerted manner to manipulate the signaling machinery of the host B-cell.

## Introduction

Gammaherpesviruses are amongst the most prevalent of human pathogens owing to their ability to establish lifelong persistent infections within their hosts [1]. Persistence is achieved through the establishment of latent infection in conventional memory B lymphocytes. To gain access to this cell type, gammaherpesviruses have developed molecular mechanisms that promote B cell activation in the absence of cognate antigen recognition [2]. Thus, host colonisation by gammaherpesvirus involves the modulation of signalling pathways triggered upon B cell receptor (BCR) activation. This strategy offers an obvious advantage to the virus since infection is not dependent on rare encounters with antigen specific naive B cells. In addition, virus driven proliferation of germinal centre (GC) B cells facilitates the amplification of viral episomes and the subsequent generation of a large pool of latent genomes in long lived memory B cells [3]. Signalling from the BCR complex is initiated when Src family kinases such as Fyn, Blk and Lyn induce the phosphorylation of immunoreceptor tyrosine-based activation motifs (ITAMs) located in the Igα and Igβ cytoplasmic tails. Tyrosine-phosphorylated ITAMs then recruit the cytoplasmic Syk protein tyrosine kinase, leading to its membrane translocation and the subsequent trans-phosphorylation of important B-cell signalling proteins such as Vav1, Vav2, phospholipase C-γ2 (PLC-γ2) and phosphatidylinositol-3 kinase (PI3K). In turn, these molecules promote the generation of a wide spectrum of intracellular signals and biological responses that are essential for the antigenic responses of B lymphocytes [4].

Several gammaherpesvirus proteins have been shown to modulate signal transduction pathways paralleled to those activated by BCR recognition of cognate antigen [2]. One such protein is latent membrane protein (LMP) 2A encoded by Epstein-Barr virus. This transmembrane protein contains two ITAM-like sequences that, upon phosphorylation, can interact with Lyn [5]. Functionally, LMP2A has been shown to drive B cell development in vivo [6], thus mimicking the presence of a functional BCR [7]. Another example is provided by the Kaposi's sarcoma-associated herpesvirus (KSHV) K1 protein. This transmembrane protein also contains a functional ITAM that mediates interactions with a large cohort of signalling proteins, including Syk, Lyn, Vav1, PLC-γ2 and PI3K [8]–[11]. Although many studies have addressed the function of these proteins [12], due to the restricted host range tropism of the human gammaherpesviruses, their role in vivo has not been fully demonstrated. Therefore, we still lack studies that can correlate the biochemical and biological properties of these viral proteins with the different biological aspects involved in the pathogenesis of those viruses in their host cells.

A suitable model to circumvent this problem is the use of murine gammaherpesvirus 68 (MHV-68), because this pathogen can infect laboratory mice [13], [14]. Despite this difference in tropism, MHV-68 can establish long term latent infection in memory B cells upon amplification of viral episomes in GC B cells [15]–[19]. During the establishment of latency by MHV-68 a selective number of viral-encoded proteins are expressed that are predicted to orchestrate biological programs in the host B cell [20]. One of these proteins is M2, a 192 amino acid-long polypeptide that bears no sequence similarity to any other known protein [21]. One of the functions of M2 is to induce the phosphorylation and activation of Vav1 and Vav2 in a BCR-independent, but Src family-dependent manner [22]. Vav1 and Vav2 work as phosphorylation-dependent guanosine nucleotide exchange factors (GEFs) for members of the Rho/Rac family [23], a group of GTPases involved in the regulation of cytoskeletal, mitogenic, and antigenic responses [24]. The activation of Vav proteins by M2 requires the formation of a trimeric complex between these two proteins and the tyrosine kinase Fyn [22], a process mediated by the recognition of a M2 proline rich region (PRR) by the SH3 domains of both Vav and Fyn proteins [22]. Interestingly, we have previously shown that the formation of this complex results also in the phosphorylation of M2 on a region that contains two tyrosine residues (Tyr 120 and Tyr 129) [22]. These results suggested that in addition to protein interaction events, the function of M2 could be modulated by phosphorylation-based signals. However, they could not exclude the possibility that this phosphorylation was a bystander defect derived from the close physical proximity of M2 and Fyn in the heteromolecular complex.

To investigate the possible implication of phosphorylation-dependent effects in the functional cycle of M2, we performed in this work experiments aimed at identifying the specific residues that were phosphorylated by Fyn on M2. In addition, we investigated the functional capabilities of M2 mutant proteins lacking different combinations of these two putative phosphorylation sites at the biochemical and signalling level. To obtain additional information about the regulatory properties of M2 in vivo, we also compared the pathogenicity of M2 mutant viruses with disrupted phosphorylation sites, with wild type MHV-68 and with recombinant viruses bearing previously reported inactive M2 mutant proteins, such as an M2 frame shift mutant [17] and a PRR-mutant version that cannot trigger the stimulation of the Fyn/Vav1/Rac1 pathway [22]. Interestingly, these experiments demonstrated that the phosphorylation of M2 on the tyrosine 120 is critical for the optimal assembly of the M2/Fyn/Vav1 complex and, perhaps more importantly, for the activation of the latency program of MHV-68 in infected mice. They also provided evidence suggesting that the action of M2 in that pathogenic program involves the manipulation of additional signalling molecules within B-cells. Taken together, these results reveal additional mechanistic aspects of the cross-talk established among M2 and elements of the B-cell signaling machinery that are crucial for the orchestration of specific biological programs linked to gammaherpesvirus latency in the host B-cell.

## Results

### Phosphorylation of M2 on Tyr120 is required for Vav1 activation

**Figure 1:**
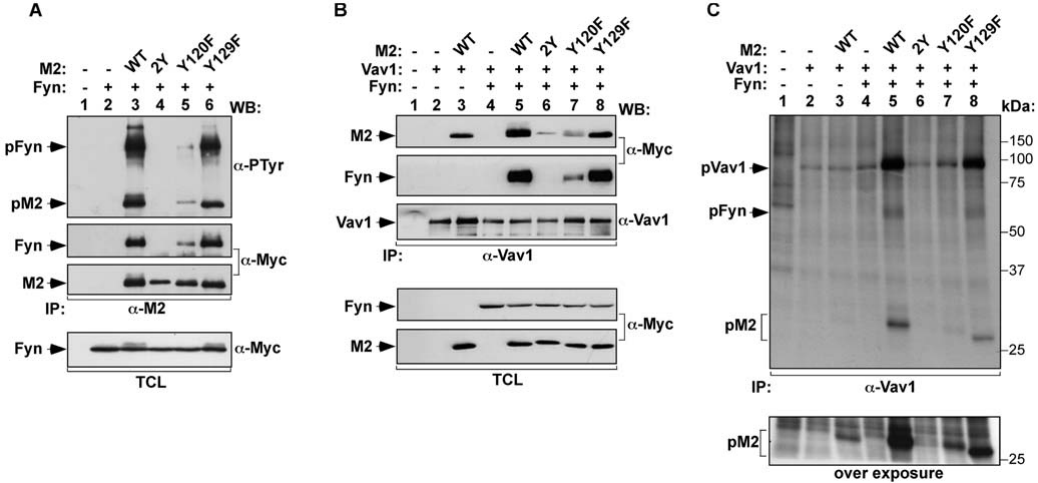
Y^120^ is the predominant tyrosine required for the formation of the M2/Vav1/Fyn complex and for M2-dependent phosphorylation of Vav1. COS1 cells were transiently transfected with plasmids encoding the indicated proteins. (A,B) Cells were lysed and clarified extracts incubated with either anti-M2 (A) or anti-Vav1 antibodies (B). Immunoprecipitates were analysed by western blot with the indicated antibodies (right side of panels). Western blot analysis of a representative aliquot of total cell lysates (TCL) confirmed expression of proteins (bottom panels). (C) Cell lysates were incubated with an anti-Vav1 antibody and the resulting immunoprecipitates subjected to an immunocomplex kinase assay exactly as described previously [22]. Lower panel is an overexposure showing phosphorylation of M2 and its mutant forms. The position of molecular weight markers is indicated on the right. −, without; +, with; α, anti; IP, immunoprecipitation; p, phosphorylated; WB, western blot.

We have previously shown that a M2 mutant protein (M2Y) containing two missense mutations on tyrosine residues 120 and 129 (the amino acid residue positions used in this study for M2 refer to the final spliced form of this protein, [25]) could not be phosphorylated by Fyn in vivo or in vitro [22], indicating that these residues were the actual Fyn targets in M2. To identify the exact acceptor site of the phosphate group, we generated two M2 mutant proteins in which either the Y120 (M2Y120F mutant) or the Y129 (M2Y129F mutant) residues were mutated to phenylalanine. To monitor the phosphorylation of these mutants, these proteins were expressed in COS1 cells in the presence of Myc-tagged Fyn, immunoprecipitated, and subjected to immunoblot analysis with anti-phosphotyrosine (PTyr) antibodies. As a control, we used COS1 expressing either the wild type or the previously described M2Y mutant version of M2 [22]. These experiments revealed a graded effect of the mutations in the total levels of M2 phosphorylation. Thus, the M2Y129F mutant displayed only marginal defects on tyrosine phosphorylation when compared to its wild type counterpart (Fig. 1A, upper panel), indicating that this site is not the main target of Fyn. In contrast, the phosphorylation of M2 was drastically reduced and totally abolished in the case of the M2Y120F and the M2Y mutant versions, respectively (Fig. 1A, upper panel). These results indicate that the main phosphorylation site of M2 is Tyr120.

To investigate the effect of the Y to F mutations in the function of M2, we used the above anti-M2 immunoprecipitates to monitor the association of this protein with Fyn. Using anti-Myc immunoblots, we observed that Fyn could co-immunoprecipitate at similar levels with both wild type M2 and the M2Y129F mutant protein. In contrast, its association with M2 was severely reduced in the case of the M2Y120F mutant and totally abolished when co-expressed with the M2Y mutant (Fig. 1A, second panel from top). As previously described [22], Fyn was also tyrosine phosphorylated in this complex as demonstrated by the detection of phospho-Fyn in the anti-M2 immunoprecipitates derived from cells expressing either the wild type or the M2Y129F versions (Fig. 1A, upper panel). These results suggest that Y120 is involved in the physical interaction of M2 with Fyn in vivo.

We examined next the role of these two M2 phosphorylation sites in the coassembly of Fyn and Vav1 and the subsequent phosphorylation of Vav1. To this end, we co-transfected COS1 cells with Vav1, Fyn and wild type M2 or M2 tyrosine mutants in the indicated combinations, and examined Vav1 immunoprecipitates for the presence of Fyn and M2 proteins (Fig. 1B). While wild type M2 and the M2Y129F mutant co-immunoprecipitated equally well with Vav1, the interaction of both M2Y and M2Y120F mutants with this GEF was significantly reduced although, unlike the case of the interaction of Fyn with M2Y, not completely abolished (Fig. 1B, top panel). Likewise, Vav1 associated optimally with Fyn when co-expressed with either wild type M2 or the M2Y129F mutant. Instead, this complex was partially or totally lost when M2 was replaced by the M2Y120F and M2Y mutants in the transfections, respectively (Fig. 1B, second panel from top). In agreement with these results, in vitro kinase assays indicated that the optimal phosphorylation of Vav1 by Fyn could be only triggered by wild type M2 and M2Y129F proteins but not by the rest of Y to F M2 mutants used in the study (Fig. 1C). Taken together, our results indicate that Y120 is the primary phosphorylation residue involved in the formation of the M2/Vav1/Fyn complex and in Vav1 phosphorylation.

### Constitutive phosphorylation of M2 in B cells

**Figure 2:**
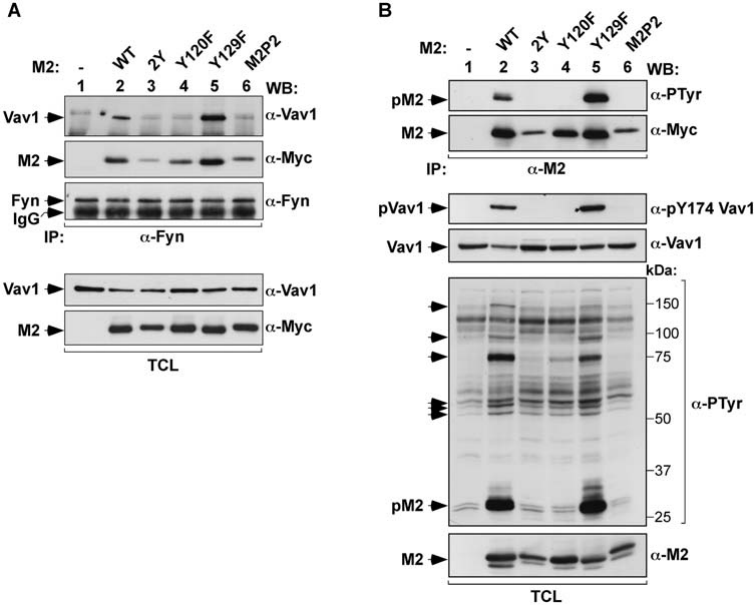
Role of the phosphotyrosine M2 motif in B-lymphocytes. (A) TCLs from A20 cells expressing the indicated proteins were incubated with anti-Fyn antibodies and subjected to western blot analysis using the indicated antibodies. As control, aliquots of the same TCLs were analyzed with anti-Vav1 and anti-Myc antibodies to reveal the levels of endogenous Vav1 and ectopic M2 proteins present in these lysates. (B) M2-transfected A20 cells were lysed and TCLs analyzed either by immunoprecipitation with anti-M2 antibodies followed by immunoblot with the indicated antibodies (two upper panels) or by direct immunoblot with the indicated antibodies (rest of panels). The mobility of M2, Fyn and Vav1 proteins is indicated by arrowheads. The mobility of M2-dependent phosphorylated cellular proteins is indicated by arrows (fifth panel from top). α, anti; IP, immunoprecipitation; p, phosphorylated; WB, western blot.

In order to address the role of M2 phosphorylation in a more physiological context, we next monitored the coassembly with Fyn and Vav1 and the phosphorylation of M2 in A20 cells, a mouse B cell lymphoma line expressing surface IgGs. To this end, M2 and its phosphotyrosine mutants were ectopically expressed in these cells. For comparative purposes, we also analyzed in these experiments the binding properties and phosphorylation of M2P2, a previously described M2 mutant protein that lacks the PRR involved in the recognition of both Vav1 and Fyn [22]. The formation of heteromolecular complexes was assessed by immunoblot analysis of Fyn immunoprecipitates for the presence of Vav1 and M2 proteins. These results indicated that Y120 is the primary phosphorylation residue involved in the formation of the M2/Vav1/Fyn complex in B cells (Fig. 2A). Next, we examined the phosphorylation levels of M2 proteins by anti-PTyr immunobloting of M2 immunoprecipitates. Under these conditions, we observed that wild type M2 was constitutively phosphorylated on tyrosine residues even in the absence of BCR stimulation (Fig. 2B, upper panel; data not shown). This constitutive phosphorylation could be due to the relatively high basal levels of activation of this cell line [26]. This phosphorylation was maintained upon mutation of the Y129 residue but abolished in the case of the replacement of the Y120 M2 residue (Fig. 2B, upper panel). As expected [22], the elimination of the proline rich region eliminated the phosphorylation of M2, as assessed by the lack of detectable phosphorylation levels in the M2P2 mutant protein (Fig. 2B, upper panel). These results indicate that the main phosphorylation site of M2 is Y120 independently of the cell type used. To analyze the impact of these mutations in the activation of Vav1 in B-cells, we monitored by immunoblot analysis the levels of phosphorylation of endogenous Vav1 on tyrosine 174 in the transfected A20 cells. Phosphorylation of this Vav1 site is a good reflection of its activation status, since it has been shown before that this posttranslational modification induces a conformational change in the Vav1 molecule that stimulates its GDP/GTP exchange activity towards Rho/Rac proteins [27]–[29]. Furthermore, it has been previously shown that M2 induces Vav1 phosphorylation on this site and the concomitant activation of the downstream Rac1 GTPase [22]. As shown in Fig. 2B, the phosphorylation of Vav1 induced by the overexpression of M2 in B-cells did not occur in the case of M2Y120F-transfected cells. As previously described, Vav1 phosphorylation was also impaired when the M2P2 mutant protein was expressed in A20 cells. In contrast, the Y129F mutation did not affect the phosphorylation of the endogenous Vav1 protein at the Y174 position (Fig. 2B).

To verify whether M2 could affect other intracellular proteins in addition to Vav1, we analyzed total cell extracts from A20 cells by anti-PTyr immunoblot to monitor the possible phosphorylation of other B-cell proteins on tyrosine residues upon overexpression of M2 and the indicated mutants. We observed that M2 and the M2Y129F mutant induced the phosphorylation of several proteins of approximately 52, 54, 56, 75, 95 (likely Vav1 itself) and 150 kDa (Fig. 2B, fifth panel from top). Interestingly, the phosphorylation of these proteins was not observed in A20 cells expressing either M2Y and/or M2P2 and significantly reduced in the case of cells expressing the M2Y120F mutations (Fig. 2, fifth panel from top). Taken together, these results show that Y120 is the predominant phosphorylated tyrosine of M2 both in lymphoid and non-hematopoietic cells. Moreover, they indicate that M2 is also able to promote the BCR-independent phosphorylation of Vav1 and an additional subset of cellular proteins. This effect is totally dependent on the presence of both the Y120 residue and the PRR of M2.

### Phosphorylated M2 binds directly to Vav1 and Fyn SH2 domains

**Figure 3:**
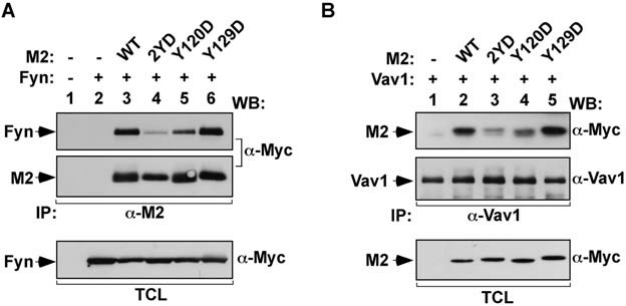
Increased association of Fyn and Vav1 to phosphorylated M2 does not involve phosphorylation-dependent conformational changes. (A,B) COS1 cells were transiently transfected with plasmids encoding the indicated proteins. After 48 h, cell extracts were obtained and subjected to immunoprecipitation with either anti-M2 (A) or anti-Vav1 (B) antibodies. Immunoprecipitates were analyzed by western blot with the indicated antibodies (right side of panels). Aliquots of the TCL used in the immunoprecipitations were analysed in parallel to confirm expression of the appropriate proteins (bottom panel). −, without; +, with; α, anti; IP, immunoprecipitation; WB, western blot.

The observation that tyrosine phosphorylation regulates the binding of Fyn and Vav1 to M2 was quite unexpected, since we had shown before that the interaction of these proteins was mediated by the M2 PRR [22]. We surmised that the involvement of the phosphotyrosine M2 motif in this heteromolecular interaction could be due to two different causes. On the one hand, it was possible that tyrosine phosphorylation could induce a conformational change in M2 that resulted in the exposure of the hidden PRR, thereby facilitating the interaction of this second motif with the M2 target proteins. On the other hand, it was also feasible that the phosphorylation sites of M2 could serve as additional docking sites for Vav1 and Fyn via interactions with the SH2 domains of these two signalling proteins. To verify the former possibility, we assessed the binding of several M2 mutants containing Y to D mutations at positions 120 and 129 to Fyn and Vav1. These mutations mimic the negative charge created by the incorporation of the phosphate group on the tyrosine residue so, if the hypothesis of the conformational change were correct, we expected that some of these mutants could enhance the binding of Fyn and Vav1 to M2. However, we found that the Y to D mutations induced effects identical to those elicited by the Y to F missense mutations, indicating that the role of this phosphorylation site was not due through conformation-dependent effects (Fig. 3A,B, top panels).

**Figure 4:**
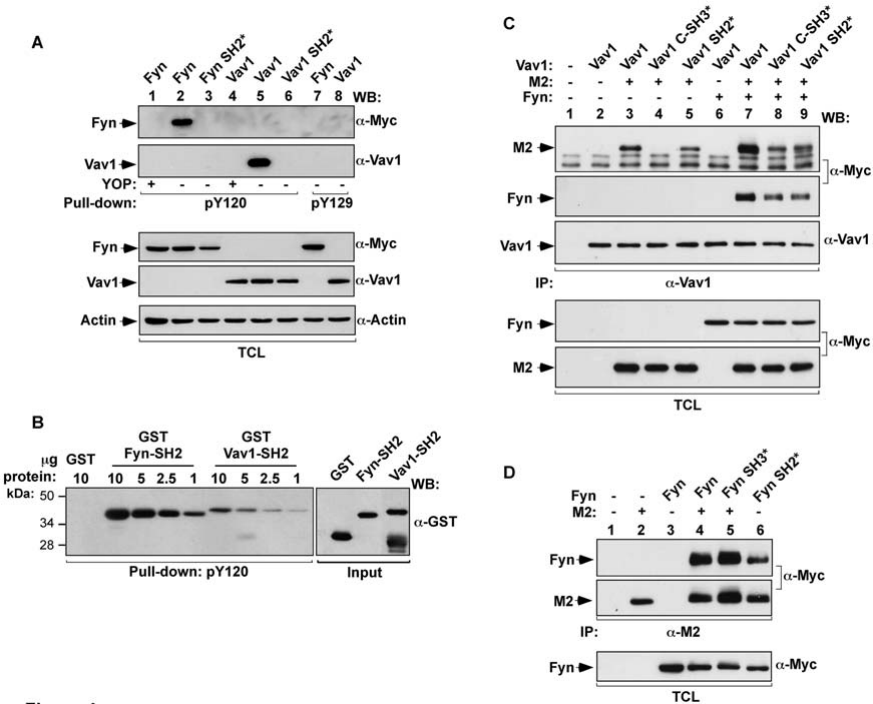
Phosphorylated M2 binds directly to SH2 domains of Fyn and Vav1. (A) COS1 cells were transiently transfected with plasmids encoding the indicated proteins (top). Cell extracts were incubated with biotinylated peptides containing M2 sequences encompassing phosphorylated (−) and YOP-mediated (+) dephosphorylated versions of residues 120 and 129. Complexes were recovered with streptavidin-coupled agarose beads and analyzed by western blot using the indicated antibodies (right side of panels) to reveal the possible association of the indicated versions of Fyn (top panel) and Vav1 (second panel from top). Western blot analysis of aliquots of TCL confirmed expression of proteins (three bottom panels). (B) The phosphorylated Y120 peptide described in (A) was incubated with increasing amounts of the indicated GST fusions proteins purified from bacteria. After binding, the peptide was recovered as indicated above and bound proteins identified by immunoblot analysis using anti-GST antibodies (upper panel on the left). Aliquots of GST proteins were analyzed by western blotting (upper panel on the right). (C,D) COS1 cells were transiently transfected with plasmids encoding the indicated proteins. Clarified lysates were incubated with anti-Vav1 (C) or anti-M2 (D) antibodies. Immunoprecipitates were analyzed by western blotting using the indicated antibodies (left). Aliquots of TCL used in the immunoprecipitations were analysed in parallel to confirm the expression of proteins used in this experiment (bottom panels). *, point mutation in the indicated domain of Fyn or Vav1; −, without; +, with; IP, immunoprecipitation; WB, western blotting.

To assess the second possibility, we first tested the hypothesis that the SH2 domains of Fyn and Vav1 could bind M2 sequences containing the phosphorylated Y120 residue. To this end, COS1 cell lysates containing either the wild type or the SH2 mutant versions of Fyn and Vav1 were subjected to pull-down experiments with phosphopeptides (pY) containing either the phosphorylated Y120 or Y129 residues. After binding, the association of Vav1 and Fyn to these peptides was determined by immunoblot analysis. As a control for the specificity of the possible interactions, we included parallel pull-downs in which peptides were dephosphorylated by the YOP phosphatase prior to the incubation with cell extracts. As shown in Figure 4A (two top panels), the pY120 peptide, but not the pY129 peptide, could bind to both Fyn and Vav1. However, this interaction was lost in the case of the SH2 mutants of Fyn and Vav1 (Fig. 4A, two top panels) or when the dephosphorylated peptide was used in the experiments (Fig. 4A, two top panels). These results indicate that pY120 residue can act as an effective docking site for Vav1 and Fyn. In order to assess if this interaction required other chaperone proteins, we repeated these experiments using the isolated SH2 domains of Vav1 and Fyn purified from *Escherichia coli* as glutathione S-transferase (GST) fusion proteins. As a negative control, we performed similar pull-down experiments with the non-chimeric GST protein. We found that the pY120 peptide could bind directly both SH2 domains, but that the SH2 domain of Fyn bound more efficiently than that of Vav1 (Fig. 4B). The phosphorylated peptide failed to bind to GST (Fig. 4B), confirming the specificity of the interactions. These differences could not be attributed to loading artefacts, given that equivalent amounts of peptide and input GST proteins were used in each pull down experiment.

To confirm that Vav1 binds to both phosphorylation sites and the PRR of M2, we next compared the association of M2 with wild type and mutant Vav1 proteins with inactivated SH2 and the C-terminal SH3 domains either in the absence or presence of Fyn. For these experiments, Vav1 N-terminal SH3 mutants were not analyzed since we have previously shown that M2 only binds to the C-terminal SH3 domain of Vav1 or Vav2 [22]. In the absence of Fyn, the inactivation of the C-terminal Vav1 SH3 domain markedly reduced the M2/Vav1 interaction (Fig. 4C, upper panel, interaction observed only after longer exposure times, data not shown), confirming our previous observations [22]. In contrast, the inactivation of the Vav1 SH2 domain affected only marginally the association of Vav1 with M2 under these conditions (Fig. 4C, upper panel). This is consistent with the low phosphorylation levels of M2 in the absence of Fyn (see above, Fig. 1C, lower panel). As expected [22], co-expression of Fyn enhanced the interaction between M2 and Vav1 (Fig. 4C, upper panel). Under these conditions, a significant level of coimmunoprecipitation was observed between M2 and SH2 and C-terminal SH3 mutants of Vav1 (Fig. 4C, upper panel). Therefore, under conditions of optimal M2 tyrosine phosphorylation, these two domains of Vav1 could efficiently establish physical contacts with the viral protein. Consistent with previous results [22], we found that Fyn formed part of the M2/Vav1 complexes (Fig. 4C, second panel from top). This association could be detected regardless of the Vav1 protein used (Fig. 4C, second panel from top), indicating that the association between Vav1 and Fyn is made indirectly via the scaffolding action of M2. As expected, Fyn also associated with the phosphorylation sites and the PRR of M2, however, in this case binding through the SH2 domain had a stronger influence (Fig.4D, upper panel). Control immunoblots showed equivalent expression levels of M2, Fyn and Vav1 proteins in the appropriate samples (Fig. 4C,D). Taken together, these results indicate that Fyn and Vav1 use two independent docking sites to associate stably with M2.

### The phosphosites and PRR of M2 contribute to the establishment of latency in GC B cells but they are not responsible for the full complement of M2 functions

**Figure 5:**
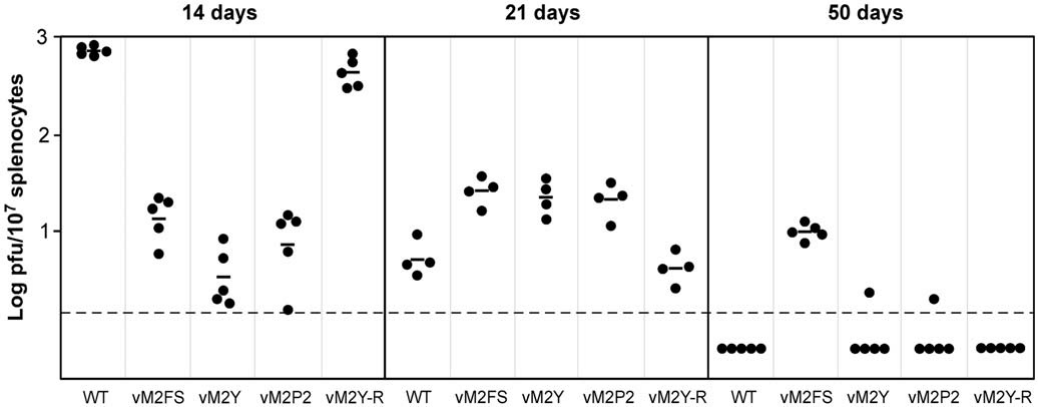
Activation of Vav proteins is necessary to the establishment of normal levels of latency in spleen. Balb/c mice were intranasally infected with viruses of the indicated viral genotypes and latent load quantified by ex-vivo reactivation. Each point represents the infectious center titre from an individual mouse. Horizontal bars indicate arithmetic means. The dashed line indicates the limit of detection of the assay.

In order to determine the functions of the identified phosphosites and the PRR of M2 in physiological context of latency in vivo, we generated MHV-68 recombinant viruses in which the *m2* gene was modified to contain the mutations in either the phosphorylation motif or the PRR (designated vM2P2, with proline residues at positions 158, 160, 163 and 167 mutated to alanine). Given that the Y120F M2 mutant still displayed some basal phosphorylation in some cell contexts (i.e., COS1 cells), we decided to use in these experiments a virus encoding the M2Y mutant (designated vM2Y, with tyrosine residues at positions 120 and 129 mutated to phenylalanine), a version of M2 that shows no detectable phosphorylation even under conditions of Fyn overexpression (see above, Fig. 1A). Moreover, this mutant totally disrupts the interaction with Fyn and the phosphorylation of B-cell proteins (see above, Figs. 1A and 2), thus ensuring the total blockage of the signals emanating from this M2 region. The DNA structures of mutant viruses were verified by PCR, DNA sequencing and examination of restriction enzyme digestion profiles of *Escherichia coli*-derived BAC DNA and PCR of reconstituted virus DNA. The stability of the introduced mutations was further checked in viruses recovered from latently infected spleens confirming the retention of the engineered point mutations. Spliced M2 transcripts could be readily detected in RNA extracted from fibroblasts infected with each of the viruses, confirming that the mutations did not affect transcription of M2 (data not shown). These viruses were used to infect mice and analysed for the previously shown functions of M2 in latency. In addition, we used in this study a previously described M2 frame shift mutant (vM2FS) that does not express this protein [17]. By using this additional virus, we wanted to distinguish whether the phosphosites and the PRR mutants were responsible for either the totality or just a subset of the functions previously ascribed to M2 during B-cell infection [17], [30]–[32]. To analyse the behaviour of these viral mutants during latency, we infected Balb/c mice with each of these viruses and monitored both the establishment and the long-term latency of MHV-68 in B cells. To this end, we subjected these animals to three independent, although complementary biological assays: *ex vivo* reactivation assays to measure latent infection in total splenocytes, flow cytometry coupled to limiting dilution and real time PCR to quantify the frequency of viral DNA positive GC B cells in spleen, and *in situ* hybridization analysis to identify virally infected cells within the spleen. Using the former assay, we observed in the case of the wild type virus the expected peak of infection at day 14 post-inoculation, with latent infection subsiding thereafter to become undetectable at day 50 post-inoculation (Fig. 5) [33]. In contrast, the vM2Y and vM2P2 viruses showed a vM2FS-like pattern of infection during the establishment of latency. This was characterised by an approximately 100-fold deficit of latent infection at day 14 post-inoculation and a subsequent increase in infectious centres by day 21 post-inoculation when compared with infections made with wild type viruses (Fig. 5). No preformed infectious virus could be detected by suspension assay of freeze-thawed spleen homogenates at any time point and for any virus analyzed, indicating that splenic infection was only latent. However, unlike the case of the vM2FS mutant virus, the vM2Y and vM2P2 viruses became undetectable during long-term latency (Fig. 5). A revertant virus (vM2Y-R) in which the *M2Y* locus was restored to wild type status did not show any defects in the course of infection, indicating that phenotypic changes observed with the M2Y mutation were intrinsic to this locus and not the consequence of mutations elsewhere in the viral genome (Fig. 5). These results indicate that the PRR and the phosphosites of M2 are responsible for engaging the cellular responses important for the establishment, but not maintenance, of latency.

To assess whether the deficit in the establishment of latency exhibited by the M2 mutant viruses reflected a phenotype in GC B cells, we determined the frequency of GC B-cells harbouring viral genomes. In mice infected with vM2Y or vM2P2, the frequency of viral genome-positive GC B cells only peaked at day 21 post-infection (Table 1). This was in contrast with the frequencies of viral genome-positive GC B cells in mice infected with wild type virus and vM2Y-R, which reached maximal levels at day 14 post-infection (Table 1). After longer post-infection periods (day 50), only mice infected with vM2FS exhibited high frequencies of viral genome-positive B cells (Table 1).

**Table 1:** Frequency of genome-positive GC B cells^a^

Days p.i.	Virus	Reciprocal frequency^b^ of viral DNA^+^ GC B cells (95% CI)	%Cells^c^	Total no. of cells^d^	No. of viral DNA-positive cells^e^
14	WT	3 (2–6)	5.7	1.1×10^7^	3,666,667
	M2FS	61 (39–142)	5.7	1.1×10^7^	180,328
	M2Y	23 (15–53)	7.8	1.6×10^7^	608,696
	M2P2	26 (17–60)	8.9	1.8×10^7^	692,308
	M2Y-R	6 (4–13)	6.8	1.4×10^7^	2,333,333
21	WT	79 (51–175)	6.0	1.2×10^7^	151,899
	M2FS	6 (4–12)	6.0	1.2×10^7^	2,000,000
	M2Y	23 (14–58)	4.3	8.6×10^6^	373,914
	M2P2	10 (7–21)	4.1	8.2×10^6^	820,000
	M2Y-R	51 (32–119)	4.9	9.8×10^6^	192,157
50	WT	148 (92–380)	2.1	4.2×10^6^	28,378
	M2FS	8 (6–17)	4.9	9.8×10^6^	1,225,000
	M2Y	128 (77–381)	1.5	3.0×10^6^	23,437
	M2P2	256 (169–527)	2.6	5.2×10^6^	20,233
	M2Y-R	78 (52–162)	1.7	3.4×10^6^	43,897

a Data were obtained from pools of at least five spleens.

b Frequencies were calculated by limiting-dilution analysis with 95% confidence intervals (CI).

c The percentage of GC B cells from total spleen was estimated by FACS analysis.

d The total number of cells was estimated from the percentage of the total spleen, based on an estimate of 2×10^8^ cells/spleen.

e The number of latently infected cells was based on the frequency of latency within GB B cells and the estimated total number of cells.

**Figure 6:**
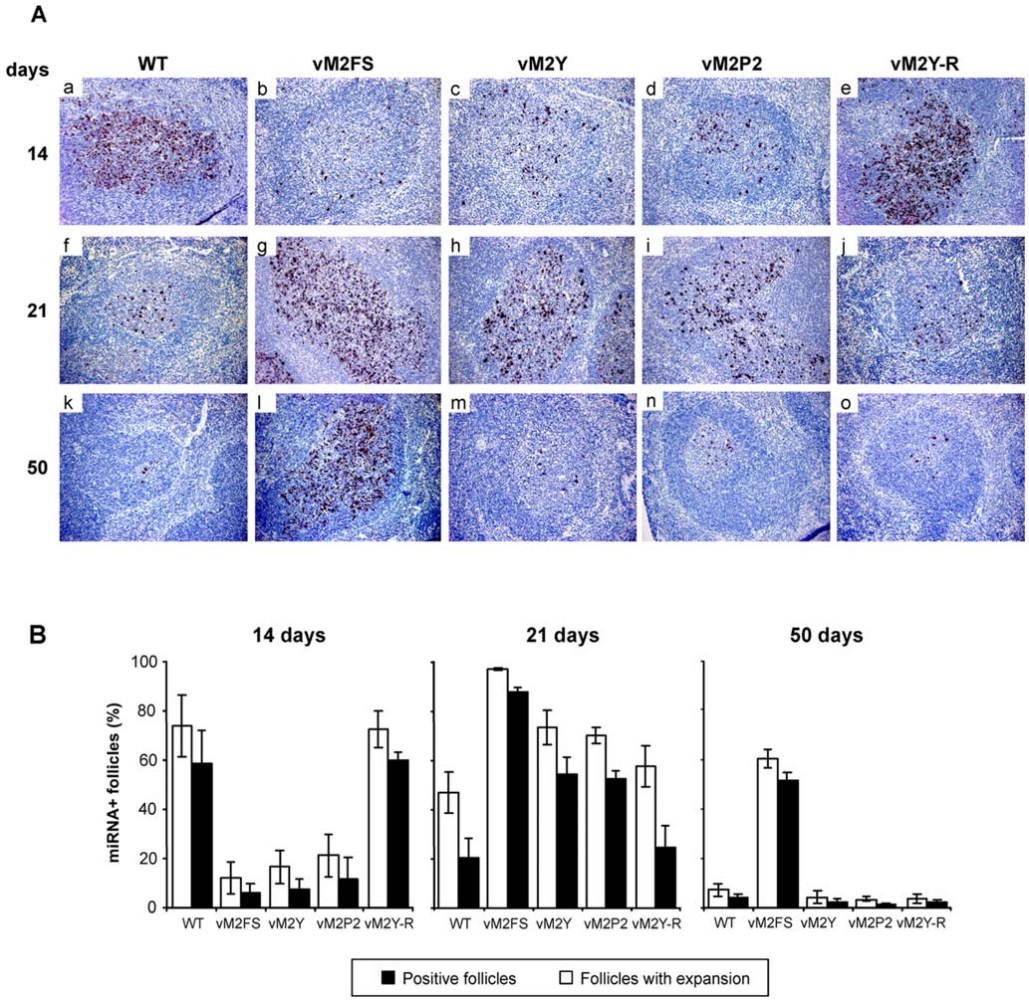
Modulation of Vav activity is required for normal kinetics of latency in GC B cells. Balb/c mice were intranasally infected with viruses of the indicated viral genotypes. Spleen sections were processed for in situ hybridization with miRNA riboprobes. (A) Representative spleen sections from each group of animals. Dark staining indicates cells positive for viral encoded miRNAs. All sections are magnified at ×200 and counter stained with haematoxylin. (B) Mean percentage ± SEM of miRNA^+^ follicles (filled bars) and positive follicles with large clusters of miRNA-positive cells (open bars). Six sections per mouse and at least four mice per group were counted at each time point. Follicles were scored positive if they contained at least one positive miRNA cell and positive for expansion if they contained more than ten miRNA positive cells.

To verify that this infection profile was linked to the residency of the virus in GC B cells, we monitored the presence of each of those viruses by detecting transcripts corresponding to the MHV-68-derived microRNAs using *in situ* hybridization, an assay that allows the analysis of the expansion and cessation kinetics of latent infection within GCs [13], [18], [34]. Mice infected with either wild type or vM2Y-R viruses showed the expected pattern of infection (Fig. 6A,B) [18]. This pattern was characterized by the detection of large clusters of infected cells within GCs at day 14 post-infection that reflect cellular proliferation and, thereby, expansion of the latently infected cell pool (Fig. 6A, panels a,e). At day 21 post-infection, we observed sharp declines in the total number of infected GCs and in the number of GCs that were associated with the presence of large clusters of latently infected cells. This pattern reflects the cessation of the virus driven GC B cell proliferation (Fig. 6A, panels f,j; and Fig. 5B). At later periods (day 50), infection became confined to a reduced number of cells scattered within secondary follicles, a phenotype that correlates with the maintenance phase of latent infection (Fig. 6A, panels k,o). The kinetics and pattern of infection in the spleens of mice inoculated with vM2FS was as previously reported [17]. Thus, after infection with this virus, maximal numbers of large clusters of infected GC B cells were reached only at day 21 post-infection (Fig. 6A, panel g). High levels of latent infection were still observed at day 50 post-infection (Fig. 6A, panel l), where almost 100% of the miRNA-positive follicles presented with large clusters of infected cells (Fig. 6B). In the case of infections by vM2Y and vM2P2, there was a reduced number of miRNA positive follicles at day 14 post-infection (Fig. 6A, panels c,d). This deficit reflected both a decreased number of infected follicles as well as a low number of positive follicles with large clusters of latently infected cells when compared to wild type virus (Fig. 6B). In this case, maximal levels of GC infection were only observed at day 21 post-infection (Fig. 6A, panels h,i). These levels declined thereafter and reached values equivalent to those observed in the case of wild type and the vM2Y-R viruses (Fig. 6A, panels m,n). Taken together, these results indicate that the PRR and the phosphotyrosine sites of M2 work coordinately during infection in vivo, where they play essential roles in the M2 functions linked to the establishment of latency within GC B-cells. Furthermore, they suggest that the functions of M2 related to latency maintenance are probably due to other motifs of this molecule, raising the possibility that separate regions of the M2 molecule regulate different branches of the pathogenicity program of MHV-68.

## Discussion

M2 is a MHV-68 encoded protein that modulates signalling pathways linked to the establishment of viral latency in B cells. M2 contributes to the latency program through the manipulation of key signal transduction pathways [22], [35]. In order to do so, M2 utilises different structural motifs present in its primary structure. For example, it has been shown that the activation of the Vav pathway requires the interaction of the M2 PRR with the C-terminal SH3 domains of Vav1 and Vav2 proteins [22]. Likewise, the M2-dependent down-modulation of the STAT pathway requires a scattered motif distributed between the central basic region and N-terminal amino acids of M2 [35], [36]. Whether other regions of M2 contribute to these or other functions remained to be determined up to now. Here, we show that a tyrosine-phosphorylated region of M2 constitutes a third M2 regulatory motif that contributes to the establishment of MHV-68 latency in B cells. This region contains two tyrosine residues (Tyr120 and 129) that are inducibly phosphorylated in ectopic systems by Fyn and constitutively phosphorylated in the A20 B-cell line. By using mutant proteins targeted in these two residues, we have demonstrated that Tyr120 is the primary phosphorylation site of M2 while Tyr129 contributes much less to the overall phosphorylation of the protein. Interestingly, we have observed that this new region and the M2 PRR need to work together in order to activate optimally the Fyn/Vav1 pathway.

The structural dissection of these two motifs indicates that the cooperative action of the phosphotyrosine residues and the PRR is not mediated by major structural changes that expose putative cryptic binding sites of the M2 molecule. Instead, we have demonstrated that these two motifs work as independent docking sites for the SH3 and SH2 domains of both Vav1 and Fyn. In agreement with this view, we have observed that the optimal binding of Fyn to M2 requires the presence of both the phosphotyrosine and PRR motifs. Furthermore, we have also shown that Vav1 proteins with non-functional C-terminal SH3 or SH2 domains can still bind to M2, although at much lower levels than those observed with the wild type protein. Supporting this dual docking site model, we have also shown that the defective interaction of the Vav1 SH3 mutant with M2 is rescued by the overexpression of Fyn and the subsequent phosphorylation of M2. The requirement of two independent motifs for the optimal binding of Vav proteins to other partners is not unprecedented. Thus, previous studies have shown that binding of Vav1 to Cbl-b requires the SH2 and SH3 regions [37] and, of particular relevance to the current study, it has been reported that the binding of Vav2 (and presumably the rest of Vav family proteins) to mDia/interacting protein requires the simultaneous engagement of a tyrosine phosphorylation motif and a PRR [38].

The study of the signalling properties of M2 and its mutants in B-cells has revealed that this protein may also use other B-cell targets. Thus, we have shown that the enforced expression of M2 in A20 cells results in the tyrosine phosphorylation of five additional proteins whose molecular weights range between 50 and 150 kDa. Consistent with our observations with Vav1 and Fyn, we have observed that the mutation of M2 in either the phosphotyrosine motif or the PRR disrupt these phosphorylation events. Likewise, we have shown that the Y120 phosphorylation residue has a much relevant biological role than the Y129 site in this cellular response. These results underscore the concept that the phosphotyrosine motif and the PRR of M2 exert intertwined actions for the optimal manipulation of the B-cell signalling machinery. At this moment, we do not know whether these additional M2-dependent phosphoproteins are direct M2 binding partners or, alternatively, downstream elements activated by either Fyn or Vav1. Preliminary experiments favour the former possibility, since we have observed by pull-down experiments that M2 phosphopeptides can bring down some of those phosphoproteins. Thus, it is likely that, as in the case of the KSHV K1 protein [9], [11], M2 could mediate the formation of a complex signalosome with a subset of B-cell signalling proteins. We are conducting currently proteomic experiments to identify these new putative M2 targets.

The presence of two interacting motifs in the M2 structure raises interesting regulatory possibilities. According to the previous model of interactions of Vav1 and Fyn with the M2 PRR, it was difficult to understand how the M2/Fyn/Vav1 complex could be assembled, since Fyn and Vav1 will be binding to the same M2 PRR. The presence of two docking sites involved in this interaction solves this problem, since it is possible that the trimeric complex will be formed through the independent binding of Vav1 and Fyn to either the phosphotyrosine motif or the PRR. Given that both proteins can bind to these two regions of M2, this trimeric complex may form in different ways. For example, Vav1 could associate with the PRR or the phosphotyrosine motif when Fyn is bound to the phosphotyrosine or PRR, respectively. In favour of this hypothesis, we have observed that Fyn can be detected in an M2-dependent manner in the anti-Vav1 immunoprecipitates regardless of whether this GEF has inactivating mutations in either the SH2 (that disrupts its association with the M2 phosphotyrosine motif) or the C-terminal SH3 domain (which blocks its association to the M2 PRR). However, we cannot exclude the possibility that the trimeric complex could be also formed with those two proteins binding simultaneously to the two M2 docking sites. This alternative mechanism of assembly is only feasible if M2 forms homodimers within the host cell. Preliminary experiments conducted in our lab indicate that this is not the case (data not shown). An issue that is still problematic according to this model of interaction is the understanding of how Fyn becomes associated with M2 in the first place. According to our present results, Fyn only binds to M2 when both the PRR and the phosphotyrosine domains are functional. If so, how is this complex formed when M2 is non-phosphorylated? To solve this signalling conundrum, we favour a model in which the association of Fyn and M2 will entail two independent, and mechanistically separable steps. In the first step, the initial binding of Fyn to M2 would be triggered by the prior trans-phosphorylation of M2 by Fyn in the absence of complex formation or, alternatively, by the binding of Fyn to M2 molecules previously phosphorylated by other protein tyrosine kinases. This trans-phosphorylation could occur in the absence of direct physical interactions since the constitutive localization of M2 in the plasma membrane makes it possible the presence of other membrane-bound kinases in the neighbourhood [22]. This model is mechanistically similar to the binding of Syk to the tyrosine-phosphorylated sequences of the Igα and Igβ ITAM regions. This initial phosphorylation step would lead to the subsequent physical interaction of Fyn to either uncomplexed M2 proteins or to the M2/Vav1 complex, a result that would trigger the subsequent phosphorylation of Vav1 by the associated kinase. In any case, it is worth mentioning that M2 can be detected in binary complexes with Vav1 and Fyn alone, indicating that M2 will display different forms of engagement during the establishment of viral latency such as free forms, binary complexes with either Vav1 or Fyn, and the trimeric M2/Vav1/Fyn complex. This spectrum of states may be even larger if the additional phosphoproteins triggered by M2 in B-cells also associate with M2 following Vav1- or Fyn-like mechanisms.

Our results with the Y to F M2 mutants also suggest that the phosphorylation levels of the wild type M2 protein could be used to induce different signalling outputs within the host cells. For instance, we have observed that the phosphorylation pattern induced by M2 in B-cells follows a gradient response, with a total absence of signal in the case of the M2Y mutant, a low but detectable signal elicited by the M2Y120F mutant, and the robust phosphorylation of B-cell proteins upon expression of the wild type M2 protein. These results suggest that if M2 undergoes different levels of phosphorylation in these two residues during the MHV-68 infection, this information could be computed by the B-cell to trigger intracellular signals of different intensity and/or to stimulate distinct, signal-dependent biological programs. This possibility would add further plasticity for the regulation of the MHV-68 pathogenic program in the host cell. In this regard, it will be interesting to generate phosphospecific antibodies to M2 in the future to monitor the phosphorylation kinetics of M2 during the different phases of MHV-68 latency in vivo.

The importance of these two M2 docking motifs for MHV-68 latency is further strengthened by the data derived from the infection of mice with MHV-68 mutant viruses harbouring different M2 mutants. This genetic strategy has confirmed in vivo the important functional role of both the phosphotyrosine and the PRR motifs for the establishment of MHV-68 latency in B cells. Moreover, the observation that the phenotypes obtained by the M2Y and M2P2 proteins are identical, further strengthens the idea regarding the coordinated action of these two docking motifs during the function of M2 in the host cell. Interestingly, the use of this genetic strategy also revealed that the infection of mice with vM2Y and vM2P2 viruses does not recapitulate the overall biological program of the M2 protein during MHV-68 latency. Thus, in contrast to what is observed with a M2 frame shift mutant virus, we have observed that the infection of animals with those viruses does not result in the induction of persistent uncontrolled proliferation of infected GC B cells. These results indicate that other regions of M2 mediate this latter stage of the MHV-68 pathogenic program. Whether this is due to additional, intrinsic functions of M2 or to indirect causes, i.e. clearance of the virus by the immune system, remains to be determined.

Collectively, our present data indicates that M2 functions as a multidocking protein that promotes the spurious, BCR-independent activation of the Vav1/Rac1 pathway and other intracellular routes. Furthermore, we have demonstrated genetically that the intracellular effects triggered by the concerted action of the phosphotyrosine and PRR M2 motifs are essential for the establishment of MHV-68 latency in GC B-cells but not for the subsequent maintenance of long term latency, suggesting the possibility that M2 could trigger distinct signalling branches that contribute independently to different stages of the MHV-68 pathogenic program. Finally, the observation that M2Y and M2P2 disrupt MHV-68 latency raise the prospect of using phosphopeptides and/or PRR-containing peptides to block latency in vivo or, alternatively, to use in pharmacological approaches to block the activity of specific signalling pathways in B lymphocyte-based diseases. Further studies linking the biochemical properties of M2 with the pathogenesis of MHV-68 should provide valuable insights into the physiological role of this pathway for gammaherpesvirus host colonization.

## Materials and Methods

### Plasmids

pCMV-Myc constructs, encoding wild type M2, M2Y and M2P2 mutants, and pcDNA3-Vav1 expression plasmid encoding wild type mouse Vav1, have been described previously [22], [39]. pCMV-Myc-Fyn, encoding wild type mouse Fyn was generated by subcloning the Fyn cDNA sequences from the corresponding pCMV-HA expression construct [22]. pCMV-Myc constructs encoding Y120F, Y129F, Y120D, Y129D and Y120,129D (2YD) M2 mutants, and pCDNA3 encoding Vav1 with an inactivating amino acid substitution (R696A) in the SH2 domain [40] or encoding Fyn with an inactivating amino acid substitution (R176K) in the SH2 [41], [42] or (W119L) in the SH3 domains [43], were generated by site directed mutagenesis using a Stratagene Quickchange kit according to the manufacturer's instructions. A pCDNA3 encoding Vav1 with an inactivating amino acid substitution (P833L) in the most C-terminal SH3 domain has been described [44]. A pGEX-2T construct encoding the SH2 domain of Vav1 has been described [45]. The sequence encoding the SH2 domain of mouse Fyn (residues 145–247) was amplified by PCR and cloned into pGEX-6P-1 expression vector (GE Healthcare). All constructs were verified by DNA sequencing.

### Antibodies and fusion proteins

A rabbit polyclonal antibody to M2 has been described before [22]. Other antibodies used in this work included phosphospecific antibodies to Vav1 phospho-Y174 [46], a monoclonal antibody against the c-Myc epitope (Invitrogen/Clontech), rabbit polyclonal antibodies to Vav1 and Fyn (Santa Cruz), an anti-phosphotyrosine monoclonal antibody (PY99, Santa Cruz) and a goat polyclonal antibody to glutathione S-transferase (GE Healthcare). GST and GST-SH2 fusion proteins were produced in *Escherichia coli* by IPTG (isopropyl-B-D-thiogalactopyranoside) induction and purified with glutathione-sepharose beads using standard procedures.

### Cells and transfections

COS1 and NIH3T3 cells were grown in Dulbecco's modified Eagle's medium (DMEM) supplemented with 10% heat inactivated foetal bovine serum, 2 mM glutamine and 100 U/ml penicillin and streptamicin. Baby hamster kidney cells (BHK-21) were cultured in Glasgow's modified Eagle's medium supplemented as described above plus 10% tryptose phosphate broth. For immunoprecipitation and pull down assays, COS1 cells were transfected with 2–4 µg of the indicated plasmids using the DEAE-dextran method. A20 B cells were propagated in RPMI 1640 medium supplemented with 10% heat inactivated foetal bovine serum, 2 mM glutamine and 100 U/ml penicillin and streptamicin. For transfection, 2×10^7^ A20 cells were electroporated (270 V, 500 µF) with 15 µg of plasmid DNA using a Bio-Rad gene pulser and incubated for 24 h in supplemented RPMI.

### Immunoprecipitation and *in vitro* kinase assays

Transfected COS1 or A20 B cells were rinsed twice in ice-cold PBS and disrupted with ice-cold lysis buffer containing 10 mM Tris-HCl pH7.4, 100 mM NaCl, 1mM NaF, 1 mM orthovanadate, 0.5% NP-40 and Cφmplete protease inhibitors. Lysates were clarified by centrifugation and incubated with 1 µg of antibodies to either M2 or Vav1 or Fyn for 2 h at 4°C. A20 B cell lysates were precleared with protein-G conjugated Sepharose beads (GE Healthcare), prior to incubation with anti-M2 serum. Immune-complexes were recovered by incubation with protein G-conjugated Sepharose beads for 45 min at 4°C. After 3 washes with ice-cold lysis buffer, proteins were eluted in reducing Laemmli's buffer, resolved by SDS-PAGE, transferred to nitrocellulose and immunoblotted with the indicated antibodies. For *in vitro* kinase assays, Vav1 immunoprecipitates were obtained as described above, washed twice in lysis buffer, and then subjected to an in vitro kinase assay as previously described [22]. Proteins were eluted in reduced Laemmli's buffer and resolved by SDS-PAGE. Gels were fixed, dried and subjected to autoradiography.

### Pull-down experiments

N-terminal biotinylated, tyrosine phosphorylated (pY) peptides corresponding to a region of M2 incorporating Y120 (SPEENIpYETANSE) and Y129 (ANSEPVpYIQPIST) were purchased from Sigma. A non-phosphorylated version of the Y120 peptide was obtained by incubation with 50 U of YOP tyrosine phosphatase (New England Biolabs), for 30 min at 30°C. The enzyme was inactivated by the addition of 1 mM orthovanadate. For pull-down experiments, 10 µg of each peptide were incubated with clarified cell lysates or purified GST proteins overnight at 4°C. Peptide complexes were recovered with 20 µl of streptavidin-conjugated Sepharose beads (GE Healthcare) for 1 h at 4°C. After 3 washes in lysis buffer, proteins were eluted from the beads in reducing Laemmli's buffer, resolved by SDS-PAGE, transferred to nitrocellulose and analysed by western blot with the indicated antibodies.

### Generation of recombinant viruses

MHV-68 vM2Y (with tyrosine residues at positions 120 and 129 mutated to phenylalanines) and vM2P2 (with proline residues at positions 158, 160, 163 and 167 mutated to alanines) viruses were generated by mutagenesis of the viral genome cloned as a bacterial artificial chromosome (BAC) [47], [48]. The following point mutations were introduced on the *M2* gene by overlapping PCR using MHV-68 genomic DNA as a template: T_4221, 4228_A in vM2Y and G_4108, 4120, 4129, 4135_C in vM2P2. PCR products were inserted into the *Hin*dIII E MHV-68 fragment cloned in the pST76K-SR shuttle plasmid [17], using *Bln*I (nt 3908) and *Xho*I (nt 5361) restriction sites. The PCR-derived regions were sequenced to confirm the integrity of the mutations. Recombinant *Hin*dIII E shuttle plasmids were transformed into an *Escherichia coli* strain (DH10B) containing the wild type MHV-68 BAC (pHA3). Following a multi-step selection procedure, recombinant BAC clones were identified by DNA sequencing. To generate a vM2Y revertant virus (vM2Y-R), the wild type *Hin*dIII E pST76K-SR shuttle plasmid was transformed into DH10B cells containing the mutant BAC genome. All viruses were reconstituted by transfection of BAC DNA into BHK-21 cells using FuGENE 6 (Roche Molecular Biochemicals). The *loxP*-flanked BAC cassette was removed by viral passage through NIH *Cre 3T3* cells and limiting dilution to obtain GFP-negative viruses.

### Analysis of recombinant viruses

Groups of 6- to 8-week old female BALB/c mice (Instituto Gulbenkian de Ciência, Portugal) were inoculated intranasally with 10^4^ p.f.u. in 20 µl of PBS under halothane anaesthesia. At 14, 21 or 50 days post-infection, spleens were removed and processed for subsequent analysis. Titres of infectious virus were determined by suspension assays of freeze-thawed spleen homogenates using BHK-21 cells. Latent virus load was quantified by using explant cocultures of single-cell suspension splenocytes with BHK-21 cells. Plates were incubated for four (suspension assays) or five (coculture assays) days, then fixed with 10% formal saline and counterstained with toluidine blue. Viral plaques were counted with a microscope. The frequency of MHV-68 genome-positive GC B cells was determined by limiting dilution combined with real-time PCR, essentially as previously described [20]. GC B cells (B220^+^; PNA^hi^) were obtained from pools of five spleens using a BD FACSAria Flow Cytometer (BD Biosciences). The purity of sorted cells was always >98%. Real-time PCR was performed on a ABI Prism 7000 Sequence Detection System (Applied Biosystems) according to the manufacturer's instructions, using the fluorescent Taqman methodology. It is important pointing out that the change in the value range in these experiments when compared with previous published work using the Light Cycler apparatus from Roche Molecular Biochemicals, is due to the higher sensitivity of the real-time PCR methodology performed here. Noteworthy, that this change in sensitivity affects the total values but not the fold difference in the frequencies of viral DNA positive cells for wild type MHV-68 when compared to vM2FS. In fact, if we compare the fold changes in the present and previous manuscripts, they are actually very similar (compare current manuscript and [20]). Primer/probe sets used were specific for the MHV-68 *M9* gene (5′ primer: GCCACGGTGGCCCTCTA; 3′ primer: CAGGCCTCCCTCCCTTTG; probe: *6-FAM*-CTTCTGTTGATCTTCC–*MGB* ). Samples were subjected to a melting step of 95°C for 10 min followed by 40 cycles of 15 s at 95°C and 1 min at 60°C. *In situ* hybridization with a digoxigenin-labelled riboprobe encompassing both MHV-68 vtRNAs and microRNAs 1 to 4 was performed on formalin-fixed, paraffin-embedded spleen sections, as previously described [49]. Probes were generated by T7 transcription of a pEH1.4 using a commercial kit from Roche Molecular Biochemicals, according to the manufacturer's instructions.
